# Identification of Estrogen Receptor *α* Antagonists from Natural Products via *In Vitro* and *In Silico* Approaches

**DOI:** 10.1155/2018/6040149

**Published:** 2018-05-10

**Authors:** Xiaocong Pang, Weiqi Fu, Jinhua Wang, De Kang, Lvjie Xu, Ying Zhao, Ai-Lin Liu, Guan-Hua Du

**Affiliations:** ^1^Institute of Materia Medica, Chinese Academy of Medical Sciences and Peking Union Medical College, Xian Nong Tan Street, Beijing 100050, China; ^2^Beijing Key Laboratory of Drug Target Research and Drug Screening, Chinese Academy of Medical Sciences and Peking Union Medical College, Beijing 100050, China; ^3^State Key Laboratory of Bioactive Substance and Function of Natural Medicines, Chinese Academy of Medical Sciences and Peking Union Medical College, Beijing 100050, China

## Abstract

Estrogen receptor *α* (ER*α*) is a successful target for ER-positive breast cancer and also reported to be relevant in many other diseases. Selective estrogen receptor modulators (SERMs) make a good therapeutic effect in clinic. Because of the drug resistance and side effects of current SERMs, the discovery of new SERMs is given more and more attention. Virtual screening is a validated method to high effectively to identify novel bioactive small molecules. Ligand-based machine learning methods and structure-based molecular docking were first performed for identification of ER*α* antagonist from in-house natural product library. Naive Bayesian and recursive partitioning models with two kinds of descriptors were built and validated based on training set, test set, and external test set and then were utilized for distinction of active and inactive compounds. Totally, 162 compounds were predicted as ER antagonists and were further evaluated by molecular docking. According to docking score, we selected 8 representative compounds for both ER*α* competitor assay and luciferase reporter gene assay. Genistein, daidzein, phloretin, ellagic acid, ursolic acid, (−)-epigallocatechin-3-gallate, kaempferol, and naringenin exhibited different levels for antagonistic activity against ER*α*. These studies validated the feasibility of machine learning methods for predicting bioactivities of ligands and provided better insight into the natural products acting as estrogen receptor modulator, which are important lead compounds for future new drug design.

## 1. Introduction

Estrogens are the prime female sex hormones and play vital roles for menstrual cycle regulation and female sexual development [1]. However, in the past twenty years, it was reported that estrogens affect both males and females physiologically, including the metabolism of carbohydrate and lipid, the skeletal homeostasis, the cardiovascular system, and the central nervous system (CNS) [2, 3]. Extensive evidence suggests that estrogens attenuate oxidative stress by preventing generation of reactive oxygen species (ROS), which was related either to regulation of ROS-generating enzymes or augmentation of ROS-eliminating mechanisms [4]. The biological effects of estrogen are regulated through estrogen receptors [5]. ER*α* (estrogen receptor *α*) has a wide distribution in the development and functioning of various organs and tissues in the body, such as the brain, bone, urogenital tract, and cardiovascular system [6, 7]. ER*α*-positive and estrogen-dependent breast cancers make up a high proportion (more than 70%) [8]. Endocrine therapy is considered as an effective treatment through blocking the ER transcription. It was also reported that ER*α* is clinically relevant in endometrial, ovarian, and other cancer types [9, 10]. Therefore, ER*α* was an ideal pharmaceutical target and a lot of ER*α* ligands have been successfully developed for ER*α*-positive breast cancer treatment [11, 12].

Selective estrogen receptor modulators (SERMs) have special action mode with ER, which act as antagonists for antibreast cancer in breast tissue, but agonists in other tissues such as the bone and cardiovascular system [13]. Therefore, SERMs, such as tamoxifen, prevent bone density loss and also benefit the cardiovascular system [14]. Although there are lots of advantages of SERMs, they still remain deficiencies. For instance, tamoxifen for long-term treatment leads to the development of endometrial cancer and drug resistance [15, 16]. Therefore, new SERMs with higher activity and fewer side effects are given more and more attention [17].

Natural products have a wide molecular diversity and range of biological properties to provide a primary resource for high-throughput screening (HTS) and virtual screening [18]. There are a lot of natural product databases for collecting constitutes of herbs, such as TCM Database@Taiwan [19], TCMSP [20], TCMID [21], CEMTDD [22], SuperToxic [23], and SuperNatural [24], providing much data for screening and mechanism of action studies [25]. It was reported that some flavonoids derived from herbs were capable of reducing bone loss and bone deterioration associated with estrogen deficiency, but they could not lead to uterotrophic effects [26]. Therefore, natural products could be potential SERMs for the treatment of cancers or other diseases.

The discovery of leads by HTS is high cost and time-consuming and demands large for labor. Therefore, virtual screening that needs less time and investment has been widely used for facilitating drug discovery [27]. Ligand-based drug design (LBDD) and structure-based drug design (SBDD) techniques were two powerful approaches to find or develop a hit or lead compound as drug candidate [28]. LBDD refers to pharmacophore, quantitative structure-activity relationship (QSAR), and machine learning techniques. SBDD includes molecular docking, molecular dynamics, and pharmacophore based on protein structure [29]. Therefore, based on the availability of the known ligands and/or target structure information, we should apply proper method to build virtual screening model. In addition, integrating the different methods together is beneficial for making up their deficiency and improving the reliability [30].

In our study, we attempted to combine the machine learning methods and molecular docking for identifying novel ER*α* ligands from in-house natural product database. Naive Bayesian (NB) and recursive partitioning (RP) models were built and validated based on training set and test set and then were utilized for classification of active and inactive from the database. These compounds predicted as ER antagonists were further evaluated by molecular docking. According to docking score and the representative structures, several compounds were selected for ER*α* competitor assay and luciferase reporter gene assay for their antagonistic activity against ER*α*. These studies provided better insight into the natural products acting as estrogen receptor modulators, which were important lead compounds for rational design of new SERMs in the future.

## 2. Materials and Methods

### 2.1. Data Collection and Preparation

There were two datasets prepared. After eliminating the duplicate structures, ER*α* antagonists with the values of IC_50_ less than 10 *μ*M were obtained from the BindingDB database [31]. In addition, corresponding decoy datasets were generated in DUD-E online database [32] with the above ER*α* antagonists. The training set and test set were generated randomly. Then inorganic salt atoms of compounds were deleted, and subsequently, the compounds were added hydrogen atoms, deprotonated strong acids, protonated strong bases, built valid three-dimensional conformation, and minimized of energy by Molecular Operating Environment (MOE). All ER*α* antagonists and decoys were marked with “1” and “−1,” respectively.

### 2.2. Molecular Descriptors

The MOE software is able to compute 186 2D descriptors as well as 148 3D molecular descriptors [33]. 2D molecular descriptors are defined to be numerical properties that can be calculated from the connection table representation of a molecule. 2D descriptors refer to notation and terminology, physical properties, subdivided surface areas, Kier & Hall connectivity and kappa shape indices, adjacency and distance matrix descriptors, pharmacophore feature descriptors, and partial charge descriptors. 3D molecular descriptors consist of potential energy descriptors, MOPAC descriptors, surface area, volume and shape descriptors, and conformation-dependent charge descriptors. Similarly, Discovery Studio 2016 (DS) was used to calculate the 2D descriptors, which were made up of AlogP, estate keys, molecular properties, molecular property counts, surface area and volume, and topological descriptors. Extended-connectivity fingerprint-6 (ECFP-6) was also calculated with this software.

### 2.3. Molecular Descriptor Selection

To avoid the complexity and increase the efficiency of models, we firstly selected the proper molecular descriptor by Pearson correlation analysis and stepwise variable selection method [34]. Pearson correlation analysis was used to delete the descriptors not remarkably associated with activity and highly associated with each other. The criterion of elimination was that descriptors with correlation coefficients with less than 0.1 were removed. In addition, when correlation coefficient between two descriptors was more than 0.9, the descriptor with a lower correlation coefficient to activity would be deleted. Then, the rest of the descriptors were selected by stepwise analysis. The initial regression equation was created by the first descriptor. Then, other descriptors were imported to the equation in tune. At the same time, every new regression equation would be subjected to a significance test for evaluating the addition of a new descriptor. For example, the new descriptor would be removed, if the regression equation was not “statistically significant.” In addition, the descriptors were also deleted when they did not conform to “statistically significant” in the equation. The process would be completed if there were no descriptors imported or deleted.

### 2.4. Machine Learning Models

#### 2.4.1. Naive Bayesian (NB) Classifier

Based on Bayes' theorem, Bayesian categorization model is a useful probabilistic classification model [35]. During a learning process, the algorithm could generate a series of Boolean features according to the input descriptors. The frequency of occurrence of each feature in the good subset was calculated in all data samples. Then, features of the sample were generated for applying the model to a particular sample, and weights for each feature were calculated through Laplacian-adjusted probability estimate, which was a relative predictor of the possibility of that sample being from the good subset. Bayesian categorization can process a great quantity of data with high efficiency and is immune to random noise. In this study, NB classifiers were carried out by DS 2016. The parameters remained their default values.

#### 2.4.2. Recursive Partitioning (RP) Classifier

RP generates decision tree to reveal the relationship between a dependent property (activity) and a set of independent properties (molecular descriptors) [36]. The input data were divided into two subsets based on a particular molecular descriptor and corresponding splitting value at each node of the decision tree. When there were no more significant nodes, the splitting process was finished. RP classifiers were established by using Discovery Studio (DS) 2016. In RP model, to avoid excessive partitioning, the minimum number of samples per node was set as 10 and the maximum tree depth was used as 20. Each class was weighted equally. The class for a node is the class with the greatest weighted sum of samples in the node. The Gini index was used as a measure of the increase in node purity as the result of a split.

#### 2.4.3. Model Performance

NB and RP classifiers with the two kinds of descriptors, and ECFP_6 was initially generated. Subsequently, 5-fold cross-validation for the training set, test set, and external test set was used to evaluate the performance of NB. Y-scrambling was also employed to prevent NB and RP performance from a result of chance correlation for the best models. Performances of NB and RP models were evaluated by calculating the true positives (TP), true negatives (TN), false negatives (FN), false positives (FP), sensitivity (SE), and specificity (SP), prediction accuracy of antagonist (Q^+^), prediction accuracy of nonantagonists (Q^−^), and Matthews correlation coefficient (MCC) [33].

(1) $$\begin{array}{c} SE=\frac{TP}{TP+FN},\\ SP=\frac{TN}{TN+FP},\\ Q^{+}=\frac{TP}{TP+FP},\\ Q^{-}=\frac{TN}{TN+FN},\\ MCC=\frac{\left(TP\times TN\right)-\left(FN\times FP\right)}{\sqrt{\left(TP+FN\right)\left(TP+FP\right)\left(TN+FN\right)\left(TN+FP\right)}}. \end{array}$$

### 2.5. Molecular Docking

Molecular docking was investigated to further study the binding mode of ER*α* and compounds predicted by NB and RP classifiers. We utilized the LibDock and CDOCKER protocol of DS 2016 for docking analysis. The crystal structure of ER*α* was obtained from the Protein Data Bank (PDB ID: 3ERT) [37]. LibDock is a useful algorithm for docking small molecules into an active receptor pocket. Primarily, a hotspot map is generated for the receptor active site which contains polar and apolar groups. This hotspot map is subsequently utilized to form favorable interactions by strictly aligning the ligand conformations. The ligand poses with top scoring are saved after a final energy-minimization step [38]. CDOCKER is the other important docking program in DS using a rigid receptor and CHARMmfield [39]. The interaction energy for each final pose of ligands with CHARMm energy was calculated, and the top scoring (most negative, thus favorable to binding) poses are retained. The structure of ER*α* firstly was prepared through removing water, adding hydrogen, and then we used clean protein module in DS to correct problems, such as nonstandard naming, protein residue connectivity, missing side-chain, or backbone atoms. The compounds also were prepared by hydrogen addition, conversion into 3D structures, pH-based ionization, and charge neutralization [40]. The original ligand, 4-hydroxytamoxifen, was selected to define the active pocket of ER*α*. Then, redocking was performed to calculate the root-mean-square deviation (RMSD) values between the docking and initial poses for validating the reliability of docking methods.

### 2.6. ER*α* Competitor Assay

The ER*α* binding affinities of representative compounds predicted as ER*α* antagonists were measured by fluorescence polarization procedure using green PolarScreen™ ER*α* Competitor Assay kit (Life Technologies, CA, United States of America) [41]. Briefly, 75 nM ER*α* together with 4.5 nM fluormone was mixed with a series of concentrations of the test compounds in the assay buffer. Then, they were incubated for 2 h in room temperature in black low volume 384-well assay plate with NBS surface (Corning, NY, United States of America) for fluorescence polarization assay. Subsequently, the detection wavelength was set at excitation wavelength 485 nm and emission wavelength 535 nm with bandwidths of 25/20 nm in EnVision Workstation version 1.7 (PerkinElmer, MA, United States of America). 2104 EnVision® Multilabel Plate Reader was performed for the measurements.

### 2.7. Luciferase Reporter Gene Assay for ER*α*

Cell transfection and luciferase activity assay were utilized to investigate the effects of the compounds on ER transcriptional activation [42]. The human breast cancer cell line MCF-7 was obtained from the Beijing Key Laboratory of Drug Target Research and Drug Screening, Chinese Academy of Medical Sciences (Beijing, China). Dulbecco's Modified Eagle's Medium (DMEM) culture medium containing 10% fetal calf serum (FBS), 100 U/mL penicillin, and 100 *μ*g/mL streptomycin was used as complete culture medium. The culture conditions were at 37°C, saturated humidity, and 5% CO_2_. The culture medium was changed every day and when the confluence of cells reached 90%, MCF-7 cells were digested with 0.25% trypsin containing 0.02% EDTA and then cultured under the same culture conditions. MCF-7 cells in logarithmic growth phase were resuspended in 3 mL complete culture medium and were seeded on 24-well plates with a density of 1 × 10^5^/well overnight. After incubation for 24 h, cells were grown to approximately 60–80% confluence and then washed by culture medium without serum. MCF-7 cells were cotransfected with the reporter plasmid pGL2-ERE3-luc. Based on the manufacturer's instructions, transfection was mediated by lipofectamine 3000 (Invitrogen). After incubation for 12 h, transfection medium was eliminated, and MCF-7 cells were treated with three concentrations of compounds for 24 h. Then, MCF-7 cells were washed with phosphate-buffered saline (PBS) and cell lysis was collected after oscillation with a low speed. Finally, the luciferase activity was determined by dual-luciferase reporter assay system (Promega) according to the product manual.

## 3. Results and Discussion

### 3.1. Chemical Space Analysis

A total of 2075 ER*α* antagonists with the values of IC_50_ less than 10 *μ*M were collected from the BindingDB database. 7000 decoy compounds were obtained from DUD-E online database. After random assignment, the training set was generated with 1556 active and 5000 inactive compounds, and the test set was made up of 519 active and 2000 inactive compounds.

**Figure 1:**
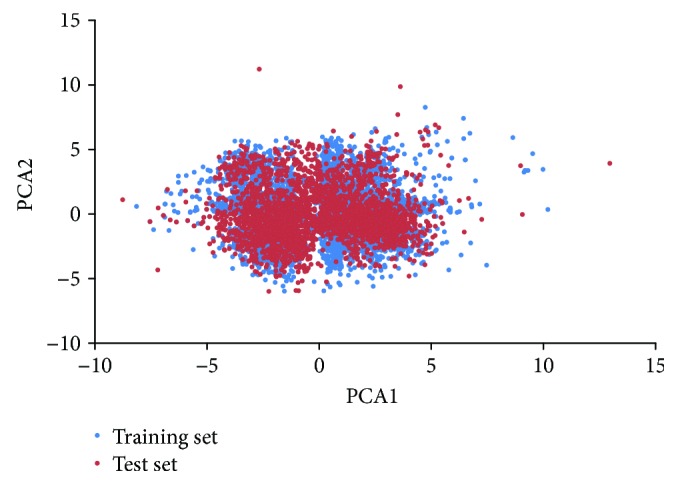
Diversity distribution of the training set and test set as described by principal component analysis (PCA).

The chemical space of the training set (compounds) and test set (2519 compounds) was investigated using principal component analysis (PCA). After Pearson correlation analysis and stepwise regression, we obtained 56 molecular descriptors (24 from Discovery Studio (DS) and 32 from MOE) (Table 1), which were used as the input variables for PCA. Chemical space analyzed by PCA was shown in Figure 1. It demonstrated that chemical space distributions were dispersive for all compounds, and most of the compounds in the test set are well within the chemical space of the training set.

### 3.2. Performance of NB and RP Models

**Figure 2:**
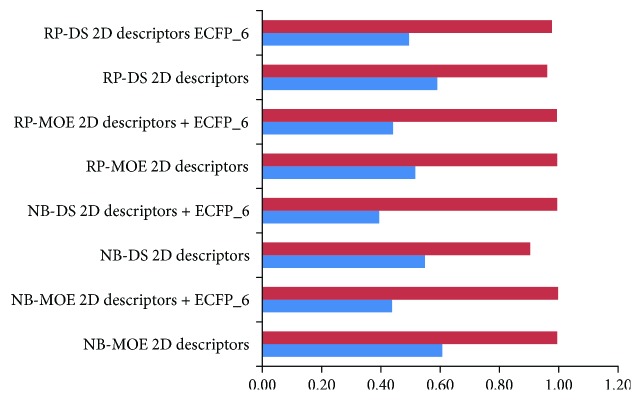
Y-scrambling for 30 times for evaluating the chance correlation possibility of naive Bayesian (NB) and recursive partitioning (RP) models by calculating Matthews correlation coefficient (MCC). The red bar represented the performance of NB and RP without Y-scrambling. The blue bar showed that the MCC values decreased after Y-scrambling.

**Figure 3:**
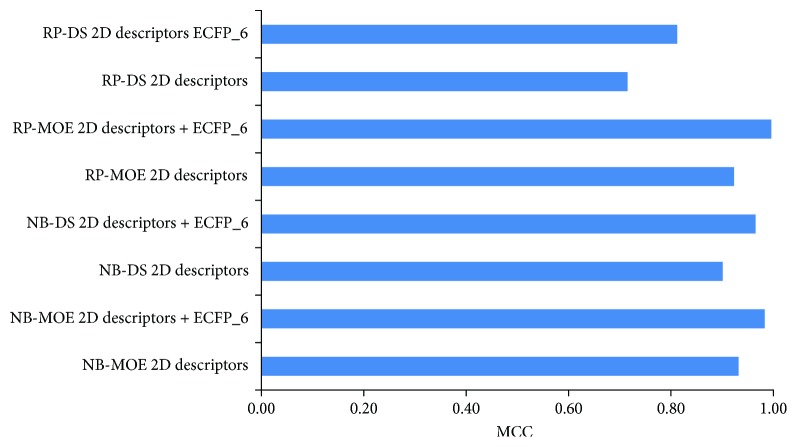
The performance of MCC value made by 8 classifiers on external test set. NB and RP MOE 2D descriptors with ECFP_6 were the most powerful models for prediction of ER*α* antagonist.

In this study, all the classification models were initially built using RP and NB classifiers with MOE and DS 2D molecular descriptors and ECFP-6. Considering the limitation of the descriptors calculated in MOE for characterizing the important substructures or molecular fragments, molecular fingerprints (ECFP-6), together with property descriptors, were used simultaneously to establish novel prediction models. Subsequently, 5-fold cross-validations for the models were performed (Table 2). The sensitivity (SE) and prediction accuracy of antagonist (Q^+^) of NB classifiers with DS 2D molecular descriptors were not favorable, but added ECFP_6, the performance was excellent with SE of 0.990 and 0.933. Y-scrambling for 30 times was as well as used to evaluate the chance correlation possibility. When the activities of compounds from the training set were disturbed, Matthews correlation coefficient (MCC) was significantly decreased, especially for NB and RP with MOE 2D descriptors in the existence of ECFP_6 (Figure 2). As shown in Table 3, DS 2D descriptors without ECFP_6 were also not better than MOE 2D descriptors in test set, which maybe DS 2D descriptors did not characterize the important substructures and molecular fragments which are critical for ER*α* antagonist. Then, compounds in external test set, which were not involved in the training and test set, were extracted from literatures published in recent years for further validation [6, 43–46]. The external test set included 20 antagonists and 50 inactive compounds.  Figure 3 suggested that NB and RP MOE 2D descriptors with ECFP_6 were the most powerful models for prediction of ER*α* antagonist. Taken together, both NB and RP models were applied for the screening of natural product database with MOE 2D descriptors and ECFP_6.

### 3.3. Good and Bad Fragments Given by Naive Bayesian Model

**Figure 4:**
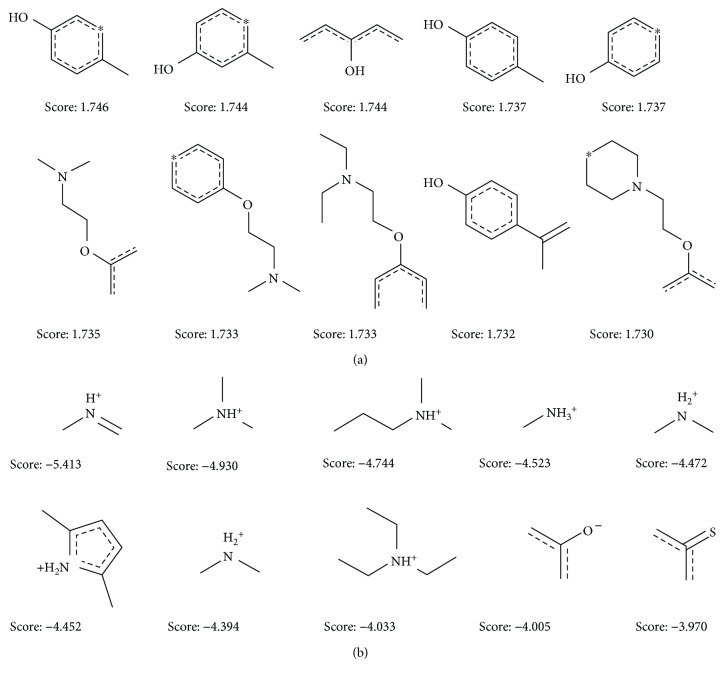
Examples of 10 good (a) and bad (b) fragments evaluated by the NB-b model. The Bayesian score (score) was given for each fragment.

ECFP_ 6, as the structural fingerprint used in Bayesian classifier, could identify key fragments or fingerprint features frequently found in two classifying groups, which provided important information for the design of ER*α* antagonist. The top 10 favorable and 10 unfavorable fragments for ER*α* binding were ranked by the Bayesian scores of the NB-b model (Figure 4). The positive fragments for ER*α* binding mainly included phenolic hydroxyl and saturated nitrogen atom. By analyzing 4-hydroxytamoxifen in the ligand-binding domain in ER*α* (PDB ID: 3ERT), we found phenolic hydroxyl could interact with Arg394 and Glu353 by forming stable hydrogen bonds. However, most fragments referring to nitrogen atoms with positive charges were observed in the negative contributions to ER*α* binding. In addition, most of unfavorable fragments contain sulfur atoms, indicating its common occurrence in many inactive ligands.

### 3.4. Virtual Screening of an In-House Natural Product Database for ER*α*

The best models (NB-b and RP-b) were applied for virtual screening of an in-house natural product database (including 13166 compounds) to identify for ER*α* lead ligands. First of all, each compound was prepared by calculating 25 descriptors (24 DS descriptors and ECFP_6) and 33 descriptors (32 DS descriptors and ECFP_6), respectively, then NB-b and RP-b were performed to evaluate the probability as ER*α* antagonists for each compound. 393 compounds were predicted as potential ER*α* antagonists by the NB-b model, while 193 compounds were predicted as active compounds against ER*α* using the RP-b model. By analyzing the overlapping part, 162 compounds were predicted as ER*α* antagonists with the two models simultaneously. Then, these compounds were further evaluated by molecular docking. The greatest advantage of LibDock is its high speed and parallel operation, which makes it suitable for large scale applications. Therefore, we used LibDock for further screening. Then, RMSD value calculated through redocking between the docking and initial poses was 1.410 Å, which suggested the reliability of LibDock methods. CDOCKER uses simulated annealing to optimize each conformation in the active site region of the acceptor, thereby making docking results more accurate. Therefore, we utilized CDOCKER to analyze the interaction of ligand and receptor. We also calculate the RMSD for CDOCKER, and the value was 1.242 Å, which validated that the docking models could be used for further docking studies. After preparation of 162 compounds, LibDock was firstly performed for quick screening. Most of these compounds could dock to the pocket of ER*α* with a wide range of LibDockScore from 27.76 to 176.61. By cluster analysis, we found the structures mainly referring to isoflavone, flavone and their glycoside, lignan, dihydrochalcone, polyphenol, catechin, and triterpenoid. To investigate the binding affinity and modes of these compounds, we selected 12 compounds with high score and representative structure. They were genistein, daidzein, phloretin, ellagic acid, ursolic acid, EGCG, kaempferol, naringenin, diosmin, naringin, silibinin, and genistein 7-O-*β*-D-glucoside. Genistein and daidzein were selected as two isoflavones, while kaempferol and naringenin were flavones. Phloretin, silibinin, ellagic acid, EGCG ((−)-epigallocatechin-3-gallate), and ursolic acid were representative of dihydrochalcone, lignin, polyphenol, catechin, and pentacyclic triterpenoid, respectively. Naringin, diosmin, and genistein 7-O-*β*-D-glucoside belonged to glycoside. Their binding affinities and modes with ER*α* were investigated as follows.

### 3.5. Binding Affinities and Modes

**Figure 5:**
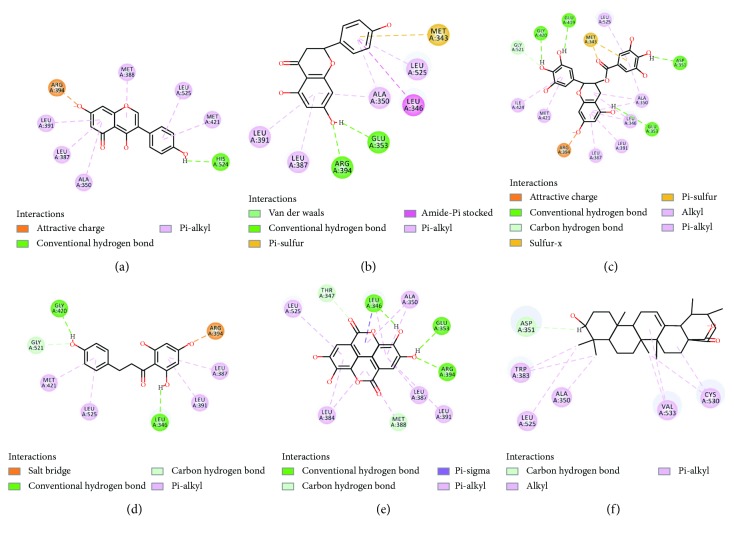
The investigation of the binding modes of 6 different skeleton structures. They were genistein (a), naringenin (b), EGCG (c), phloretin (d), ellagic acid (e), and ursolic acid (f), which belong to isoflavone, flavone, catechin, dihydrochalcone, polyphenol, and triterpenoid.

The binding affinities of 12 compounds were measured by green PolarScreen ER*α* Competitor Assay. Among these compounds, diosmin, naringin, silibinin, and genistein 7-O-*β*-D-glucoside showed no significant binding affinity with the value of IC_50_ beyond 10 *μ*M.  Table 4 summarizes IC_50_ values of the other 8 compounds tested. Genistein had the highest inhibitory activity with IC_50_ value of 29.38 ± 6.13 nM. But when genistein formed glycoside, the binding ability would be decreased sharply. The IC_50_ value of daidzein was 107.62 ± 9.38 nM, not better than genistein. The two flavone compounds, kaempferol and naringenin, showed moderate affinity with the values of IC_50_ of 316.67 ± 14.33 nM and 967.54 ± 70.95 nM, respectively, which suggested that the affinity of isoflavone was better than that of flavone. The common amino acid residues for the four compounds binding to ER*α* included Arg394 and Glu353 via conventional hydrogen bond/attractive charge and Leu387 and Leu391 via pi-alkyl interaction (Figure 5). Arg394 and Glu353 were also the key amino acid residues for 4-hydroxytamoxifen interacting ER*α*. Although -CDOCKER energy and -CDOCKER interaction energy did not fluctuate significantly, the scores of isoflavones were a little better than those of flavones. In addition, compelling evidences suggested that isoflavones had multiple beneficial effects on breast and prostate cancers, menopausal symptoms, neurodegeneration, and so on [47]. Ellagic acid, phloretin, and EGCG did not belong to flavonoid skeleton, but they also had a better binding affinity. The values of IC_50_ were equally matched with 74.55 ± 24.24 *μ*M, 62.61 ± 9.34 *μ*M, and 66.01 ± 11.59 *μ*M, respectively. Polyphenol structures of EGCG and ellagic acid form a lot of hydrogen bonds, which lead to high binding affinity, but lack of hydroxyl group, ursolic acid had weak interaction with ER*α*. One phenolic hydroxyl group of phloretin forms salt bridge with Arg394, which was different from other compounds (Figure 5). Therefore, phenolic hydroxyl groups and conjugated structures made these natural products high binding affinities, which were associated with the results of good fragments given by Bayesian model.

### 3.6. Antiestrogenic Effects

**Figure 6:**
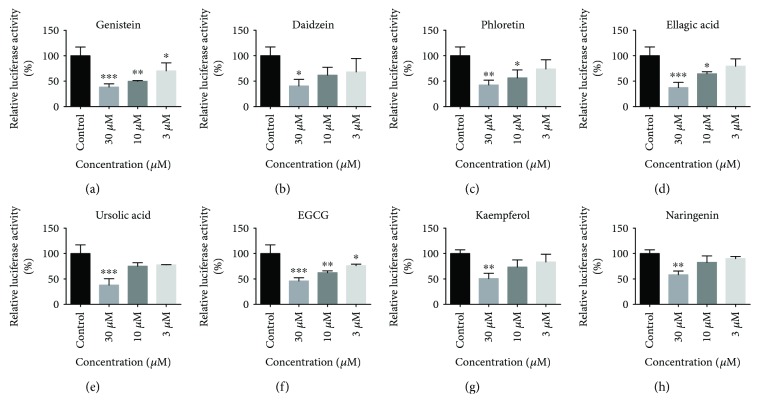
Antiestrogenic effects of 8 natural products in the ER*α* transactivation assay using MCF-7 cells transiently transfected with pERE-TATA-Luc. Cells were treated with the tested chemicals at a series of concentrations. The values represent the mean ± SD of three independent experiments and are presented as the percentage of the response, with the control defined as 100%. ^∗^*P* < 0.01, ^∗∗^*P* < 0.05, and ^∗∗∗^*P* < 0.001.

To explore whether 8 compounds have endocrine disrupting effects mediated by ER*α*, we determined their antiestrogenic activities using luciferase reporter gene assay systems.  Figure 6 showed their effects on the expression of ER*α*. We found that genistein could decrease the expression of the ER*α* remarkably at a dose-dependent manner. Recent studies suggested genistein had an important role in the suppression of breast cancer via the competition of phytoestrogen with natural estrogens, declination of their bioavailability, and inhibition of cancer cell growth [48]. It was also reported that genistein can significantly attenuate oxidative stress by modulating the JNK3-mediated apoptosis, ERK1/2-mediated autophagy, and TNF*α*-associated inflammatory pathways [49]. Although daidzein was also attributed to isoflavone, the antagonist activity was lower than that of genistein. Ellagic acid, a plant-derived polyphenol, could also decrease the expression of ER*α* at high and medium concentrations. It was also reported that ellagic acid had an influence on ER*α*-mediated signaling pathway in many kinds of cancer cell [50]. We provided the direct proof that ellagic acid had a strong interaction with ER*α* and inhibited its expression. Phloretin is a natural dihydrochalcone and displays antioxidative and anti-inflammatory activity [51]. We firstly reported that phloretin could bind to ER*α* directly and regulated the expression of ER*α* in MCF-7 cells in a dose-dependent way. EGCG is one of the most potent and the most studied green tea catechins. There was much evidence about the involvement of EGCG for antioxidant and anti-inflammatory effects. Our results suggested that ER*α* was a target of EGCG to exhibit multiple pharmacological activities. Ursolic acid generated antiestrogenic effects only at high dose. Kaempferol and naringenin, as two flavone compounds, showed weak antiestrogenic effects, which was associated with the binding affinity results. It proved again that the antagonistic activity against ER*α* of isoflavone was superior to that of flavone.

## 4. Conclusion

In our study, we integrate the ligand- and structure-based methods for identification of ER*α* antagonist from in-house natural product library for the first time. As a result, 162 compounds were predicted as ER antagonists by NB-b and RP-d models, which were further evaluated by molecular docking, and 12 compounds were selected for activity validation. Based on the ER*α* competitor assay and luciferase reporter gene assay, we found 8 compounds exhibited antagonistic activity against ER*α*, including genistein, daidzein, phloretin, ellagic acid, ursolic acid, EGCG, kaempferol, and naringenin. The affinity of isoflavone was superior to flavone, and genistein had the highest inhibitory activity. However, the binding ability of genistein would be decreased significantly when it formed glycoside. It was also first reported that ellagic acid, phloretin, and EGCG could directly bind to the active pocket of ER*α* with high affinity due to their phenolic hydroxyl group and conjugated structure. Therefore, natural products offer a rich resource for ER*α* antagonist. In addition, virtual screening method for hit discovery and lead optimization would accelerate new drug discovery and increase efficiency and decrease the cost of the drug development process, contributing to more effective, safe drugs entering into market much at a quick pace.

## Figures and Tables

**Table 1:** 56 molecular descriptors selected by the Pearson correlation analysis and stepwise regression.

Descriptor class	Numbers of descriptors	Descriptors
DS descriptors	24	PEOE_VSA_FNEG,PEOE_RPC+,a_base,a_ICM,a_nBr,a_nCl,a_nN,a_nO,a_nS,ast_violation,b_rotR,BCUT_SMR_0,BCUT_SMR_1,chi1_C,chiral_u,density,GCUT_PEOE_1,GCUT_SLOGP_1,GCUT_SMR_0,PEOE_VSA_NEG,PEOE_VSA_POS,PEOE_VSA+ 3,PEOE_VSA-0,PEOE_VSA-1,PEOE_VSA-3,PEOE_VSA-6, radius,reactive,rings,SMR_VSA0,vdw_vol,vsa_don
MOE descriptors	32	a_donacc,a_ICM,a_nCl,a_nN,a_nO,a_nS,b_rotR,BCUT_SMR_1,chi1_C,chiral_u,density,FCharge,GCUT_PEOE_1,GCUT_SLOGP_1,PEOE_RPC,PEOE_RPC+,PEOE_VSA_FNEG,PEOE_VSA_FPOL,PEOE_VSA_NEG,PEOE_VSA_POS,PEOE_VSA+ 1,PEOE_VSA-0,PEOE_VSA-1,PEOE_VSA-3,PEOE_VSA-6,rings,SlogP, SlogP_VSA7, SMR_VSA0,SMR_VSA7,vdw_vol,vsa_don

**Table 2:** Performance of Bayesian and recursive partitioning models and their 5-fold cross-validation results.

Model	TP	FN	FP	TN	SE	SP	MCC	Q^+^	Q^−^
NB-a	1091	35	223	5206	0.969	0.959	0.874	0.830	0.993
NB-b	1119	8	21	5408	0.993	0.996	0.985	0.982	0.999
NB-c	749	316	455	5037	0.704	0.917	0.591	0.622	0.941
NB-d	1054	11	75	5416	0.990	0.986	0.953	0.933	0.998
RP-a	1113	13	51	5379	0.988	0.991	0.966	0.956	0.998
RP-b	1111	15	49	5381	0.987	0.991	0.966	0.958	0.997
RP-c	1007	57	177	5314	0.946	0.968	0.876	0.850	0.989
RP-d	1022	42	95	5396	0.960	0.983	0.925	0.915	0.992

Note: NB-a: NB model with MOE 2D descriptors; NB-b: NB model with MOE 2D descriptors + ECFP_6; NB-c: NB model with DS 2D descriptors; NB-d: NB model with DS 2D descriptors + ECFP_6; RP-a: RP model with MOE 2D descriptors; RP-b: RP model with MOE 2D descriptors + ECFP_6; RP-c: RP model with DS 2D descriptors; RP-d: RP model with DS 2D descriptors + ECFP_6.

**Table 3:** Performance of Bayesian and recursive partitioning models on the test set.

Model	TP	FN	FP	TN	SE	SP	MCC	Q^+^	Q^−^
NB-a	426	7	68	2018	0.985	0.967	0.904	0.862	0.997
NB-b	425	8	27	2059	0.981	0.987	0.952	0.941	0.996
NB-c	346	62	356	1756	0.849	0.832	0.559	0.493	0.966
NB-d	389	19	40	2071	0.954	0.981	0.917	0.907	0.991
RP-a	426	8	31	2055	0.983	0.985	0.948	0.932	0.996
RP-b	427	6	29	2057	0.987	0.986	0.953	0.936	0.997
RP-c	379	54	81	2005	0.875	0.961	0.817	0.824	0.974
RP-d	401	33	35	2051	0.925	0.983	0.906	0.920	0.984

Note: NB-a: NB model with MOE 2D descriptors; NB-b: NB model with MOE 2D descriptors + ECFP_6; NB-c: NB model with DS 2D descriptors; NB-d: NB model with DS 2D descriptors + ECFP_6; RP-a: RP model with MOE 2D descriptors; RP-b: RP model with MOE 2D descriptors + ECFP_6; RP-c: RP model with DS 2D descriptors; RP-d: RP model with DS 2D descriptors + ECFP_6.

**Table 4:** IC_50_ values (nM) of 8 representative compounds as ER*α* antagonist from natural products and their binding affinity evaluated by Discovery Studio 2016.

Chemical	IC_50_ (nM)	-CDOCKER_ENERGY	-CDOCKER_INTERACTION_ENERGY
Estradiol	7.38 ± 0.80	32.60	42.1106
Genistein	29.38 ± 6.13	31.63	45.9033
Daidzein	107.62 ± 9.38	38.321	42.813
Phloretin	74.55 ± 24.24	44.56	49.7287
Ellagic acid	62.61 ± 9.34	27.54	40.3217
EGCG	66.01 ± 11.59	46.72	64.262
Ursolic acid	977.38 ± 125.30	−65.38	24.1527
Kaempferol	316.67 ± 14.33	30.31	41.5717
Naringenin	967.54 ± 70.95	30.08	38.021

## Data Availability

The data used to support the findings of this study are available from the corresponding author upon request.
